# Composition and Diversity of Gut Bacterial Community in Different Life Stages of *Osmia excavata* Alfken (Hymenoptera: Megachilidae)

**DOI:** 10.3390/microorganisms12081709

**Published:** 2024-08-19

**Authors:** Guangzhao Wang, Guiping Wang, Yixiang Ma, Zhaoyun Lv, Yinwei You, Pengtao Ma, Yi Yu

**Affiliations:** 1 Institute of Plant Protection, Shandong Academy of Agricultural Sciences, Jinan 250100, China; wangguangzhao3675@163.com (G.W.); wangguiping1018@126.com (G.W.); myx4362@163.com (Y.M.); yyw30000@163.com (Y.Y.); 2 Shandong Institute of Sericulture, Yantai 264000, China; lzhy67188@sina.com; 3 School of Life Sciences, Yantai University, Yantai 264005, China; ptma@ytu.edu.cn

**Keywords:** *Osmia excavata*, gut bacteria, community, life stage, solitary bee, 16S rRNA, pollinator

## Abstract

*Osmia excavata* is an excellent pollinator in nature and plays a vital role in the conservation of agro-ecosystems and food security. Given the important role of the gut bacterial community in host health and regulation of host growth and development, using 16S rRNA gene sequencing data, the present study explored the composition of the gut bacterial community and its diversity at different life stages (eggs, young larvae, old larvae, young pupae, old pupae, and 1-day-old adults in cocoons) of the solitary bee *Osmia excavata*. The results showed that the core phyla in the gut of *O. excavata* at different life stages were Proteobacteria, Firmicutes, Bacteroidota, and Actinobacteriota, and the core genera were *Sodalis*, *Tyzzerella*, and *Ralstonia*. The highest intestinal bacterial diversity was found in the Egg period, and the lowest bacterial alpha diversity was found in the 1-day-old Adult period; the bacterial diversity of *O. excavata* showed a process of decreasing, as it was growing from the Egg period to the 1-day-old Adult period. Our study found that *O. excavata* undergoes a significant change in the structure of the gut flora when it grows from the young pupae to old pupae stage, a period of growth that coincides with the process of cocooning and isolation from the external environment after food depletion. This suggests that food and environmental factors are key contributors to the structure of the bacterial community in the gut of solitary bees.

## 1. Introduction

The microbiota of insects includes bacteria, fungi, viruses, archaea, and protozoa; of these, bacteria are present in the gut of almost all insects and are usually the most abundant microorganisms [1,2,3]. The gut microbiota has attracted significant research attention because of its close relationship with the host. Gut microorganisms are interdependent on, and regulate, the host and play an important role in maintaining host health. Alterations in the gut flora of insects can affect the host in terms of nutrient metabolism, immune defence, antioxidant function, and drug resistance [4,5,6]. For example, the gut microbiota of the honeybee *Apis mellifera* promotes host weight gain via bacterial metabolism and hormonal signalling [7]; locusts contain cellulase-secreting intestinal flora, providing them with a high intestinal cellulose-degrading activity and enabling them to use cellulose from grasses as their energy source [8]; the extracellular symbiont *Enterococcus mundtii* of *Spodoptera littoralis* selectively removes pathogens from the host gut by secreting a stabilising bacteriocin, promoting normal development of the gut microbiota, reducing the risk of infection through the gut, and enhancing host health [9]; and in the silkworm *Bombyx mori*, *Stenotrophomonas* increases the amount of essential amino acids in the gut, which strengthen host fitness and enable the larvae to avoid the detrimental effects of the insecticide chlorpyrifos [10].

The functions of the gut microbiota depend on its composition and structure, which show dynamic changes throughout the life stages of the host. Although insects have a simple gut microbiota compared to that of mammals, their microbiota also changes dynamically throughout their life stages [11]. During moulting and pupation in holometabolous insects, the intestinal epithelium is also shed, accompanied by the loss of the gut microbiota; the gut is then reinoculated with gut microbes [12,13]. Insects that live in colonies or are eusocial, such as termites, ants, some wasps, and bees, share living space and exhibit social interactions, such as oral or faecal feeding, which allow gut microbes to spread and be maintained within populations, either indirectly or directly [14,15,16]. Among the social insects, the gut microbiota of *A. mellifera* has been extensively studied [17], with research showing that the gut of adult worker bees contains a distinctive and specialised microbiota that is dominated by only nine bacterial species clusters [18]. Given the importance of gut microbes for mammals, including humans, and the difficulties associated with their experimental manipulation, Zheng et al. proposed that, because of its intrinsic gut microbial properties as well as established methods used to cultivate and genetically manipulate core gut bacterial species, *A. mellifera* could be used as a model insect for the study of gut microbial communities, providing an experimentally tractable model system [19]. The association of gut microbes with social insects suggests that they have coevolved [20]. However, there are also insects that are classified as solitary, with limited opportunities to transfer gut microbes between conspecifics because females often abandon their eggs after oviposition [6]. For example, larval *Osmia cornifrons* are thought to alter the microbial community of the pollen and nectar supply within their nest chamber as they feed; thus, the healthy development of solitary bees might be related to the diversity of pollen bacteria in their nests, as well as colonisation by plant microbes [21].

*Osmia excavata* Alfken is an excellent pollinator of deciduous fruit trees in northern China and is characterised by low temperature resistance, high pollination efficiency, easy management, and low application costs; thus, it has been widely used to pollinate fruits, vegetables, and other crops in recent years, significantly improving the fruiting rate and yield of cherries and apples [22,23,24]. *O. excavata* is a solitary pollinator which has one generation per year. Female *O. excavata* usually nest in reeds or holes in the walls of buildings, where they create multiple ‘cell-like’ nesting chambers from the inside out using soil and lay one egg on a pollen mass in each chamber, where they develop individually [25,26,27]. *O. excavata* has a life cycle comprising a 3–4-day egg stage, 20–25-day larval stage, 25–30-day pupal stage, and 30–45-day adult activity stage; it metamorphoses into an adult inside the cocoon during August–September, where it remains to overwinter in an diapause state and then breaks out of the cocoon during the second half of March the following year [28]. Through 16S rRNA full-length sequencing of the intestinal tract and soil and food in nests, Liu et al. found that the intestinal bacterial communities of *O. excavata* larvae were similar in different regions in China; they also found that the environment around the nest, including temperature, light, precipitation, soil moisture, and pH, can affect the gut microbiota of larval *O. excavata* by influencing the nesting materials and nectar source plants [29].

Most of the studies on *O. excavata* have focussed on its biological characteristics and pollination, with less focus on its gut microbiology because of its long life stages and the difficulty of observing the adults given the length of time they spend inside the cocoon. Thus, in this study, we analysed the composition and diversity of the gut microbiota of the different life stages of *O. excavata* by sequencing the 16S rRNA V3–V4 variable region of its microbiota using Illumina NovaSeq second-generation high-throughput sequencing. This comprehensive analysis of the composition and diversity of the bacterial community throughout the life stages of *O. excavata* provides a scientific basis and research directions for further study of the intestinal flora of solitary bees, as well as their conservation.

## 2. Materials and Methods

### 2.1. Experimental Insects

*O. excavata* is widely used as a pollinating insect for fruit trees and is available for purchase. In May 2022, we purchased a large number of egg-stage *O. excavata* from Yantai Bifeng Agricultural Science and Technology Co., Yantai, China. *O. excavata* is a solitary insect, and its parents prepare plenty of food for their offspring in the nesting chamber where they live, and their growth and development do not require human management. In nature, the company places a large number of handmade plastic tubes to facilitate the nesting of *O. excavata*, which is collected and sold when the eggs are laid, and the nest is complete.

From May to November 2022, the eggs were reared in individual plastic tubes through the following developmental stages: young larvae (Larva. Y), old larvae (Larva. O), young pupae (Pupa. Y), old pupae (Pupa. O), and 1-day-old adults in cocoons (Adult). We defined ~3-day-old larvae as young larvae and ~20-day-old larvae as old larvae; pupae with creamy-white bodies and white-pigmented eyes dissected from cocoons ~5 days after pupation were defined as young pupae, and pupae with black bodies and black-pigmented eyes dissected from cocoons ~25 days after pupation were defined as old pupae. We sampled and preserved *O. excavata* from each developmental stage as follows: the equipment needed for sampling, such as tweezers, scissors, blotting paper, and so on, were sterilised in advance. On an ultra-clean bench, scissors were used to dismantle the nesting tubes to remove the *O. excavata*, which was then surface-disinfected with 75% alcohol twice for 3 min each, followed by aseptic water twice, again for 3 min each time. Each *O. excavata* was then placed in sterilised 1.5 mL centrifuge tubes; given the size of *O. excavata* differed at each development stage, 20 eggs were added per centrifuge tube and five individuals were added per centrifuge tube for the Larva. Y, Larva. O, Pupa. Y, and Pupa. O stages. For adults in 1-day-old cocoons, the intact intestinal tissue was removed under aseptic conditions and subjected to two surface disinfections with 75% alcohol followed by two surface disinfections with sterile water. The intestinal tissue from ten adults was then placed in a 1.5 mL centrifuge tube. There were five replicates per developmental stage. All samples were snap-frozen under liquid nitrogen and stored at –80 °C until use.

### 2.2. DNA Extraction and PCR Amplification of 16S rDNA

Sample genomic DNA was extracted using the CTAB method [30]. The purity and concentration of DNA were then detected using 1% agarose gel electrophoresis. An appropriate amount of sample DNA was placed in a centrifuge tube and diluted to 1 ng/μL using sterile water. PCR reactions were performed using primers 341F: (5′-CCTAYGGGRBGCASCAG-3′) and 806R: (5′-GGACTACNNGGGGTATCTAAT-3′) using a 16S rRNA sequencing PCR reaction system as follows: 15 μL Phusion^®^ High-Fidelity PCR Master Mix (New England Biolabs, Ipswich, MA, USA), 0.2 μM primers, and 10 ng of genomic DNA template. The PCR reaction comprised the following: predenaturation at 98 °C for 1 min; denaturation at 98 °C for 10 s, annealing at 50 °C for 30 s, extension at 70 °C for 30 s for a total of 30 cycles; and a final extension at 72 °C for 5 min. After the PCR amplification products were detected by 2.0% agarose gel electrophoresis, the products were recovered using a Universal DNA Purification and Recovery Kit (TianGen, Biotech (Bejing) Co., (Beijing, China), DP214), and the library was constructed using the NEB Next^®^ Ultra™ II FS DNA PCR-free Library Prep Kit (New England Biolabs, Ipswich, MA, USA, E7430L). The resulting library was quantified by Qubit and Q-PCR, followed by NovaSeq 6000 (Illumina, San Diego, CA, USA) for PE250 online sequencing.

### 2.3. Bioinformatics Analysis

Using FLASH (Version 1.2.11), the reads of each sample were spliced, resulting in spliced Raw Tag data (Raw Tag) [31]. The spliced Raw Tags were strictly filtered using fastp (Version 0.23.1) to obtain high-quality Tags (Clean Tags) [32]. These were compared using the SILVA 138.1 database to detect chimeric sequences, which were then removed to obtain the final Effective Tags [33]. Noise reduction was performed using the DADA2 module of QIIME2 (Version QIIME2-202002) to obtain the final amplicon sequence variants (ASVs) as well as the feature list [34]. Species annotation was performed using the classify-sklearn algorithm of QIIME2 (Version QIIME2-202002) and the SILVA 138.1 database, sequences annotated to chloroplasts and mitochondria were removed, and bar charts of compositional abundance were plotted for each species.

Linear discriminant analysis (LDA) effect size (LEfSe) was used to identify intestinal bacterial taxa with significant differences among host stages. This method uses the nonparametric Kruskal–Wallis test with default settings in a rank-sum test to identify biomarkers [35]. Differences between the alpha diversity indices of the groups were analysed by plotting dilution curves for each species and calculating the Chao1, Shannon, and Simpson indices using QIIME2; unweighted UniFrac distances were calculated using QIIME2, and the extent of the differences between the samples and groups was analysed by nonmetric multidimensional scaling (NMDS) analysis and principal coordinate analysis (PCoA).

## 3. Results

### 3.1. Characteristics of the Sequencing Data

In total, 2,541,661 raw data reads were measured from all 30 samples, with 2,212,326 valid reads obtained after quality control (quality control efficiency of 87.04%). The sequences were clustered into ASVs with 100% similarity, resulting in 4,391 ASVs. Taxonomic annotation using the SILVA 138.1 database resulted in annotations of 51 phyla, 112 class, 227 orders, 352 families, and 750 genera across the life stages of *O. excavata* (Table 1). Comparison of the number of ASVs annotated from different life stages of *O. excavata* indicated that the mean number of ASVs annotated to the Egg and Pupa. Y stages was similar and much higher compared to the other four life stages (Larva. Y, Larva. O, Pupa. O, and adult). The total number of species annotated to different taxonomic orders was highest during the Egg stage but lowest during the Pupa. O stage.

### 3.2. Composition of Intestinal Microbiota

**Figure 1:**
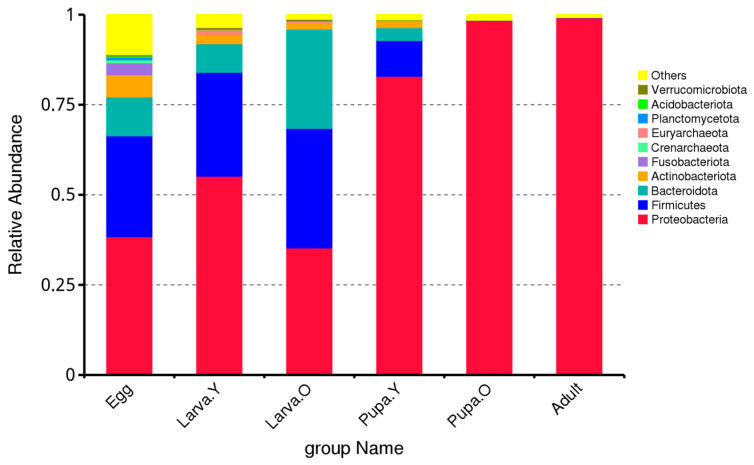
Relative abundance of intestinal microbiota at the phylum level in different life stages of *Osmia excavata*.

The top-10 phyla present in the gut microbiota across the life stages of *O. excavata* were Proteobacteria, Firmicutes, Bacteroidota, Actinobacteriota, Fusobacteriota, Crenarchaeota, Planctomycetota, Acidobacteriota, and Verrucomicrobiota. However, there were significant differences in terms of their relative abundance between the different life stages of *O. excavata* (Figure 1). For example, the relative abundance of Proteobacteria was highest in Adult and Pupa. O (99.14% and 98.27%, respectively) and lowest in Egg and Larva. O (38.41% and 35.29%, respectively); that of Firmicutes was highest in Larva. O (33.17%) and lowest in Adult and Pupa. O (0.02% and 0.16%, respectively); that of Bacteroidota was highest in Larva. O (27.56%) and lowest in Pupa. O and Adult (0.02% and 0.06%, respectively); that of Actinobacteriota was highest in Egg (6.19%) and lowest in Adult (0.08%); and that of Fusobacteriota was highest in Egg (3.33%) and relative abundance was minimal or absent during Pupa. O and Adult. Despite being in the top-10 phyla in the microbiota across the life stages of *O. excavata*, the percentage abundances of Crenarchaeota, Euryarchaeota, Planctomycetota, Acidobacteriota, and Verrucomicrobiota were all < 1% in each of the life stages. Thus, the most dominant phyla in the intestines of different life stages of *O. excavata* were, in descending order, Proteobacteria, Firmicutes, Bacteroidota, and Actinobacteriota (Figure 1). Proteobacteria, Firmicutes, and Bacteroidota predominate during Egg and Larva and play a central role in the *O. excavata* intestine; Proteobacteria predominate during Pupa and Adult and play a central role in the *O. excavata* intestine.

**Figure 2:**
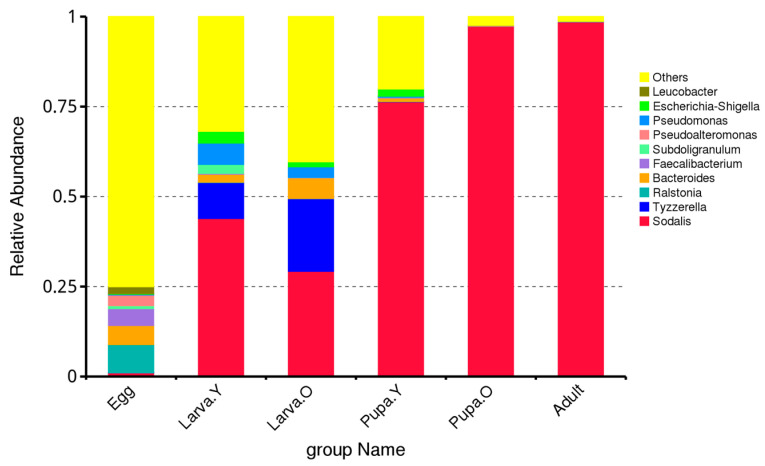
Relative abundance of intestinal microbiota at the genus level in different life stages of *Osmia excavata*.

At the genus level, the gut microbiota of the different life stages of *O. excavata* mainly comprised the following: *Sodalis*, *Tyzzerella*, *Ralstonia*, *Bacteroides*, *Faecalibacterium*, *Subdoligranulum*, *Pseudoalteromonas*, *Pseudomonas*, *Escherichia-Shigella*, and *Leucobacter*. However, there were significant differences in terms of their relative abundance between the different life stages of *O. excavata* (Figure 2). For example, *Sodalis* was most abundant in Adult and Pupa. O (98.56% and 97.35%, respectively) and least abundant during the Egg stage (1.04%); that of *Tyzzerella* was highest in Larva. O (20.18%), with little or no relative abundance in Pupa. O or Adult; that of *Ralstonia* was highest in Egg (7.86%) and lowest in Larva. O (0.05%); that of *Bacteroides* was highest during Larva. O (5.72%), with minimal or no relative abundance in Adult; that of *Faecalibacterium* was highest in Egg (4.69%), with minimal or no relative abundance in Pupa. O and Adult; that of *Subdoligranulum* was highest in Larva. Y (2.52%), with minimal or no relative abundance in Pupa. O and Adult; that of *Pseudoalteromonas* was highest in Egg (2.95%), with minimal or no relative abundance in Larva. Y and Larva. O; and that of *Pseudomonas* was highest in Egg (5.93%) and lowest in Pupa. O (0.02%).

Thus, the predominant genera in the gut microbiota of different life stages of *O. excavata* were *Sodalis*, *Tyzzerella*, and *Ralstonia*. During the Egg period, the *Ralstonia* genus dominated; during the Larva period, the genera *Sodalis* and *Tyzzerella* dominated; during the Pupa and Adult periods, the genus *Sodalis* dominated (Figure 2).

**Figure 3:**
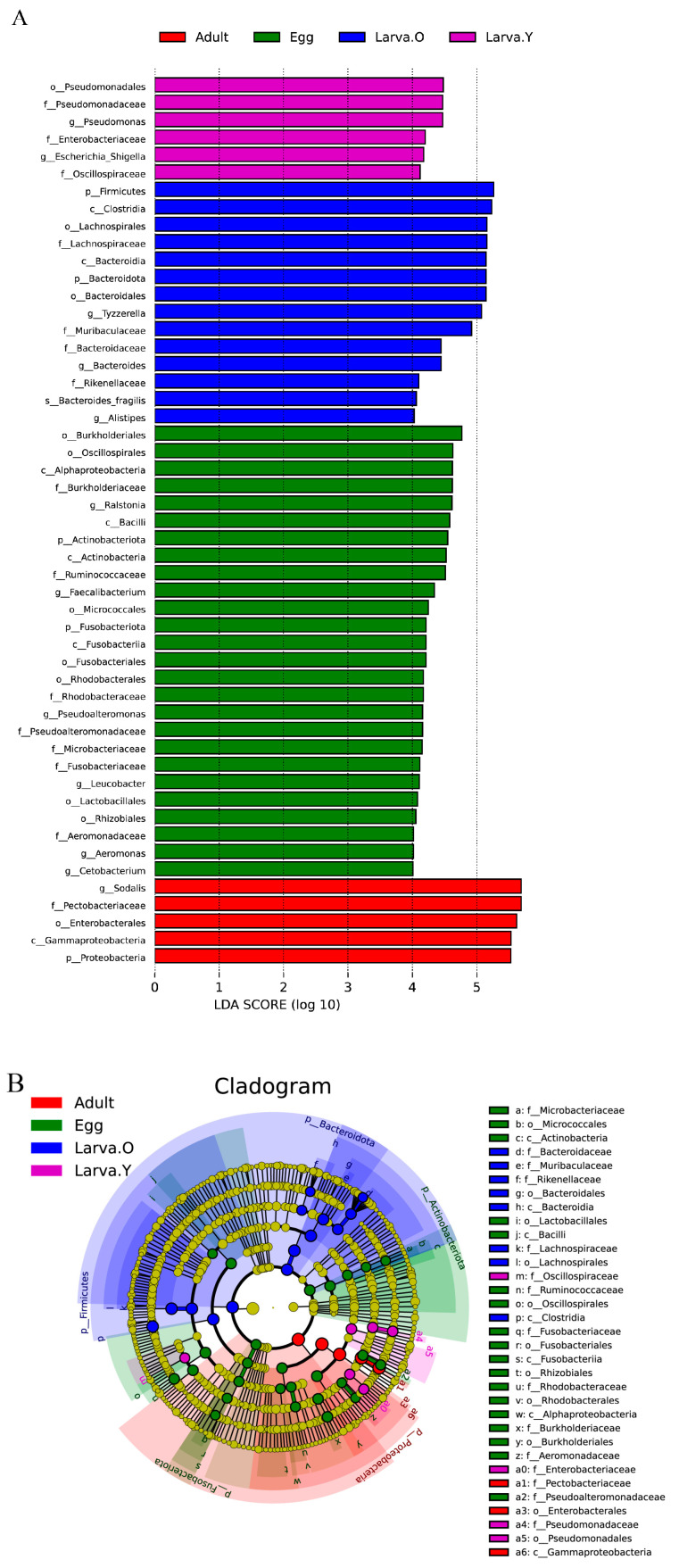
LEfSe analysis showing significant differences in microbial taxa at the phylum to genus level in different life stages of *Osmia excavata*. (**A**) LEfSe bar graphs showing the gut flora at different life stages with LDA scores > 4.0. The higher the LDA score, the more the bacterial taxon contributes to the variation. (**B**) LEfSe branching diagram showing the phylogenetic distribution of intestinal microbiota at different life stages. Yellow nodes represent microbial taxa that do not differ significantly between life stages, whereas other coloured nodes represent microbial taxa that are significantly enriched at these life stages.

Linear discriminant analysis (LDA) and effect size (LEfSe) analyses were performed to further identify specific bacterial taxa that were differentially abundant at the phylum to genus level at the different life stages of *O. excavata* (Figure 3). Only groups with LDA scores > 4.0 were analysed between the different life stages. The analysis showed that the bacterial tax in the Egg stage were mainly concentrated and enriched in two phylum (Actinobacteriota and Fusobacteriota), four classes (Alphaproteobacteria, Bacilli, Actinobacteria, and Fusobacteriia), seven orders (Burkholderiales, Oscillospirales, Micrococcales, Fusobacteriales, Rhodobacterales, Lactobacillales, and Rhizobiales), seven family (Burkholderiaceae, Ruminococcaceae, Rhodobacteraceae, Pseudoalteromonadaceae, Microbacteriaceae, Fusobacteriaceae, and Aeromonadaceae), and six genera (*Ralstonia*, *Faecalibacterium*, *Pseudoalteromonas*, *Leucobacter*, *Aeromonas,* and *Cetobacterium*). Larva. Y bacterial taxa were mainly concentrated and enriched in one order (Pseudomonadales), three family (Pseudomonadaceae, Enterobacteriaceae, and Oscillospiraceae), and two genera (*Pseudomonas* and *Escherichia_Shigella*). Larva. O bacterial taxa were mainly concentrated and enriched in two phyla (Firmicutes and Bacteroidota), two classes (Clostridia and Bacteroidia), three families (*Lachnospiraceae*, *Muribaculaceae*, and *Bacteroidaceae*), and three genera (*Tyzzerella*, *Bacteroides*, and *Alistipes*), whereas Pupa. O was not significantly enriched in bacteria. By contrast, the bacterial taxa in the Adult stage were concentrated in one phylum (Proteobacteria), one class (Gammaproteobacteria), one order (Enterobacterales), one family (Pectobacteriaceae), and one genus (*Sodalis*).

LEfSe analyses showed that most of the dominant flora in the phylum-to-genera hierarchy in the different life stages of *O. excavata* was concentrated in the Egg period and that the number of significantly enriched bacteria in the gut was similar in the Larva. Y and Adult periods; significantly enriched bacteria did not exist in the Pupa period (Figure 3).

### 3.3. Formatting of Mathematical Components

#### 3.3.1. Alpha Diversity Analysis

**Figure 4:**
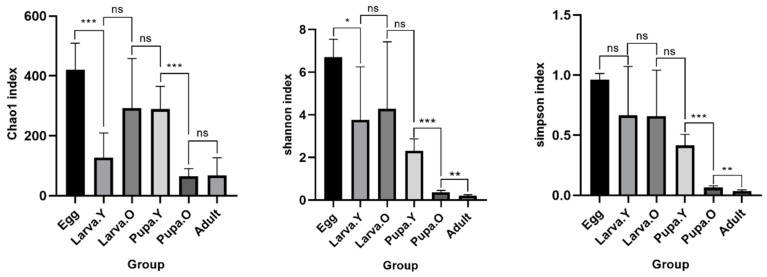
Alpha diversity of gut microbiota at different life stages of *Osmia excavata*. Asterisks indicate significant differences between different samples. ns, *p* > 0.05, * *p* < 0.05, ** *p* < 0.01, *** *p* < 0.001. The *y*-axis and *x*-axis represent the diversity index value and group, respectively.

Based on the number of total ASVs, the Chao1, Shannon, and Simpson indices were calculated to provide a measure of alpha diversity (Figure 4). There was variability in the number of bacterial taxa in different life stages of *O. excavata*, being highest in the Egg stage and significantly higher than in the Larva. Y stage. There was no significant difference in the number of species among the stages Larva. Y, Larva. O, and Pupa, followed by a highly significant increase in the number of taxa in Pupa. Y compared to Pupa. O, but no significant difference between Pupa. O and Adult. Based on the Shannon and Simpson indices, species community richness and evenness were highest in the Egg stage and lowest in the Adult stage, with no significant differences among the stages Larva. Y, Larva. O, and Pupa. Y.

Alpha diversity analysis showed that among the different life stages of *O. excavata*, the Egg period is rich in species types and has the highest number of species, while the Adult period is simple and has the lowest number of species types, and there was no significant differential change in the type and number of species in the gut from the period of growth of Larva. Y to Pupa. Y; the highest intestinal bacterial alpha diversity was found in the Egg period, and the lowest bacterial alpha diversity was found in the Adult period, and the bacterial diversity of *O. excavata* showed a process of decreasing, as it was growing from the Egg period to the Adult period.

#### 3.3.2. Beta Diversity Analysis

**Figure 5:**
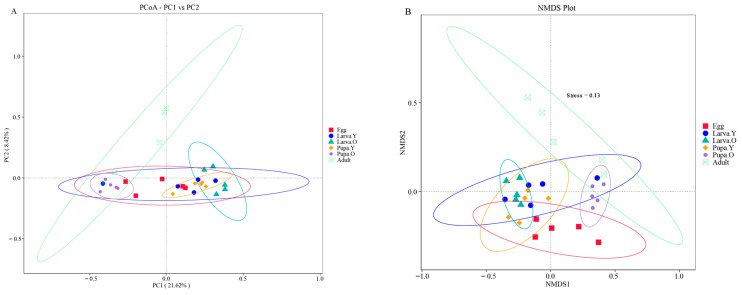
PCoA (**A**) and NMDS (**B**) analyses based on unweighted UniFrac distance.

Gut microbiota analyses of the different life stages of *O. excavata* were performed based on Unweighted UniFrac distances from PCoA and NMDS. PCoA showed that the composition of the gut flora differed among the different life stages of *O. excavata* (Figure 5A). The structural complexity of the gut flora in the Egg stage was similar to all other stages; the microbial composition structure was similar among Larva. Y, Larva. O, and Pupa. Y and between Pupa. O and Adult. Significant changes in the composition of the gut microbiota were revealed during the period of growth from Pupa. Y to Pupa. Y. NMDS analysis showed that Larva. Y, Larva. O, and Pupa. Y clustered together, as did Pupa. O and Adult, in agreement with the PCoA results (Figure 5B). In the PCoA analysis, PC1 accounted for 21.62% of the total variance and PC2 accounted for 8.42%. In NMDS, stress = 0.13, indicating reliable grouping and sampling.

The Beta diversity analysis showed that the composition of the gut flora was similar among Larva. Y, Larva. O, and Pupa. Y and between Pupa. O and Adult. However, the structure of the gut bacterial community in Larva. Y, Larva. O, and Pupa. Y has significant changes compared with Pupa. O and Adult.

## 4. Discussion

Solitary bees are important pollinators of crops and many types of flowering plant, collecting and storing pollen and nectar as food for their developing offspring. Microorganisms, including bacteria and fungi, have an important role to play in the healthy growth of solitary bees, among other organisms, including humans. This study investigated for the first time the gut microbial community of different life stages of the solitary bee *O. excavata*. In all life stages, the dominant phyla were Proteobacteria, Firmicutes, Bacteroidota, and Actinobacteriota, and the dominant genera were *Sodalis*, *Tyzzerella*, and *Ralstonia* (Figure 1 and Figure 2). The relative abundance of Proteobacteria and *Sodalis* changed significantly between the Larva. O stage to the Pupa. Y stage. At this developmental stage, all the pollen and nectar resources collected by the parent bee have been consumed, and the larva begins to grow and encase itself in a cocoon, known as the Pupa. Y stage. Thus, the significant changes in the relative abundance of Proteobacteria and *Sodali* in the gut appear to be highly correlated with this growth. Previous research reported that the bacterial genus *Sodalis* acts as endosymbiotic bacteria in a range of insects, including lice, tsetse flies, *Cicadella viridis*, and solitary bees, with varying functional roles in symbiosis [36]. *Sodalis glosinidius*, an endosymbiotic bacterium of tsetse fly, is present both intercellularly and intracellularly in tsetse tissues, including the haemolymph, fat body, and midgut, and enhances the susceptibility of tsetse to trypanosome infections by modulating the immune system of the flies [37]. *Sodalis* was also identified following sequencing of the bacterial 16S rRNA gene of the leafcutting bee *Megachile rotundata* [38]. Bacteria with cyanobacteria in the gut of pollinating insects are of great interest, and it will be annotated to species of Cyanobacteria and chloroplast in the species annotation library. Currently, research on intestinal cyanobacteria is in its infancy, with the phylum Melainabacteria representing the only nonphotosynthetic cyanobacterial member found in the intestines of humans and other animals [39]. It was found that SURIMI had the highest diversity of microbial community succession during the early stages of fermentation with cyanobacteria represented by Streptophyta [40]. Thus, we hypothesise that the bacterial genus *Sodalis* acts as an endosymbiotic bacterium that inhabits *O. excavata* maternal oocytes and uses them as a transmission vector to spread to the resulting offspring, supporting the healthy development of *O. excavata* by modulating its immune system. However, how *Sodalis* spread and function in *O. excavata* needs to be further investigated.

LEfSe analyses revealed that most of the dominant flora at the phylum to genus level occurred in the Egg stage (Figure 3); similarly, Firmicutes, Bacteroidota, and *Bacteroides* have been reported to be prevalent in the intestinal tracts of other insects, such as *Holotrichia parallela* [41], *Dendroctonus valens* [42], *Dendroctonus rhizophagus* [43]. The class Bacilli, enriched in the Egg period, and Enterobacteriaceae, enriched in the Larva. Y period, respectively, have been extensively studied. Previous work on the gut microbes of the different life stages of the stag beetle *Phalacrognathus muelleri* found that Bacilli appeared during the most advanced larval development stage [44]. Members of the Enterobacteriaceae have been found to ferment glucose and degrade sucrose to provide nutrients to their host [45]. By contrast, Pupa. Y and Pupa. O were not significantly enriched for bacteria. Given that *O. excavata* exhibits significant growth from the Larva. O to Adult stage, accompanied by reductions in the amount of significantly enriched bacteria, we hypothesise that these changes are related to the process of complete metamorphosis in *O. excavata*, whereby the intestinal epithelium is fully shed and, hence, so is the accompanying microbiota.

The larvae of solitary bees develop in separate chambers and, generally, females of solitary bees place the prepared pollen mass in the hive for consumption by their offspring, which supports larval growth and development. In addition, the female bees release fungi and bacteria associated with food spoilage and larval disease during the preparation of her offspring’s food in order to protect the healthy growth of the larvae [46]. In our study, alpha diversity was highest during the Egg period, and then it progressively decreased during development, when it was lowest in the Adult stage (Figure 4). We hypothesise that the Egg stage is a period of vulnerability for *O. excavata*; by passing on endosymbiotic bacteria from their bodies after laying their eggs, *O. excavata* females ensure the healthy development of the egg, reflected by the highest level of bacterial alpha diversity during this stage. The intestinal flora is thought to originate from the environment and diet in addition to commensal bacteria passed on from the mother [47]. The high larval diversity during the Larva to Adult period of growth may have originated from the food pollen mass prepared by the mother of *O. excavata*, which contained specific nutrients, resulting in a high alpha diversity [48]. Interestingly, the pollen mass prepared by the parent of *O. excavata* for the development of the progeny was consumed just as it developed into the pupal stage; this may have led to a decline in α-diversity as food was reduced and disappeared. The study finds that the healthy development of *O. cornifrons* larvae was associated with pollen bacterial diversity as well as colonisation by plant pathogens [21]. In addition, the insect gut microbiota has an important role in enabling the host to digest nutrients; genome-based studies showed that the presence of the bacterium *Gilliamella apicola* in honeybees enabled them to potentially digest complex carbohydrates (pectins from pollen cell walls) which could otherwise not be digested [49].

Beta diversity analyses showed that the species community structure was similar in the gut during the Larva. Y, Larva. O, and Pupa. Y stages and during the Pupa. O and Adult stages, although there were greater differences in the community structure of Larva. Y, Larva. O, and Pupa. Y stages compared to Pupa. O and Adult stages (Figure 5). Although the reason for this phenomenon is unknown, it might be related to the different functions of the gut microbiota during these different life stages: from Larva. Y to Pupa. Y, the function of the gut microbiota is biassed towards the growth and development of each individual; from Pupa. O to Adult, its function is biassed toward the construction of the gut organs after moulting and preparation for adulthood [48]. In addition, the Larva. Y, Larva. O, and Pupa. Y stages clustered closely together in the beta diversity analyses, as did the Pupa. O and Adult stages. This suggests that colonisation of the core microbiota at different developmental stages of *O. excavata* is a dynamic process, but once established, the composition remains relatively stable.

There are significant differences between the gut microbiota of social and solitary bees as a result of various differences, with social behaviour being one of the most important factors [28,50]. Previous work showed that bee social behaviour affects the abundance of the symbiont *Sodalis* (Enterobacteriaceae), causing it to be eliminated in eusocial bees, whereas it was more likely to be symbiotic in solitary bees [36]. Similarly, the current study found that *Sodalis* was a dominant genus within *O. excavata*, with extremely high relative abundance. However, the differences between the gut microbiota of eusocial bees and solitary bees, and the reasons for them, need to be explored further.

## 5. Conclusions

Our study reveals the composition, bacterial community structure, and diversity of *O. excavata* gut bacteria at different life stages. The core phyla are Proteobacteria, Firmicutes, Bacteroidota, and Actinobacteriota, and the core genera are *Sodalis*, *Tyzzerella*, and *Ralstonia*. The composition of the gut flora was similar among Larva. Y, Larva. O, and Pupa. Y and between Pupa. O and Adult; however, the structure of the gut bacterial community in Larva. Y, Larva. O, and Pupa. Y underwent significant changes compared with Pupa. O and Adult. The highest intestinal bacterial diversity was found in the Egg period, and the lowest bacterial alpha diversity was found in the Adult period, and the bacterial diversity of *O. excavata* showed a process of decreasing, as it was growing from the Egg period to the Adult period. Therefore, this study provides a basis for further understanding the role of gut microbiota in the developmental ecology of *O. excavata* and contributes to a better understanding of the interactions between gut microbes and their hosts. Future studies will build on these high-throughput sequencing results with more in-depth bioinformatics analyses and by providing insights into the role of the gut microbiota in their *O. excavata* hosts.

## Figures and Tables

**Table 1:** Results of high-throughput sequencing of the 16S rRNA gene of intestinal microbiota from different life stages of *Osmia excavata*.

Sample	Raw Reads	Valid Reads	No. of ASV	No. of Taxa				
				Phylum	Class	Order	Family	Genus
Egg. 1	92119	78673	305	12	19	40	71	122
Egg. 2	95943	83567	447	13	21	56	112	182
Egg. 3	92388	76185	513	18	25	64	115	178
Egg. 4	96365	81838	399	27	43	75	115	173
Egg. 5	91832	75047	418	24	30	49	71	67
Larva. Y1	87118	82088	47	2	2	2	2	2
Larva. Y2	84499	80444	136	15	20	41	53	68
Larva. Y3	80427	74899	179	13	20	38	52	73
Larva. Y4	77534	64460	75	10	16	24	35	37
Larva. Y5	83092	56875	215	13	20	44	62	89
Larva. O1	75106	71895	191	10	16	24	29	32
Larva. O2	84323	75774	190	9	13	26	33	44
Larva. O3	78404	67311	444	14	20	42	60	95
Larva. O4	75693	67772	312	12	19	31	48	81
Larva. O5	81790	68266	514	14	21	42	57	99
Pupa. Y1	83228	52882	578	15	26	47	80	111
Pupa. Y2	84809	75380	370	13	23	34	57	67
Pupa. Y3	80597	55972	288	9	12	22	45	65
Pupa. Y4	80301	57459	291	8	11	20	37	56
Pupa. Y5	83433	75739	400	9	14	30	50	78
Pupa. O1	87192	84738	92	3	3	3	4	3
Pupa. O2	91959	87005	122	5	5	9	14	12
Pupa. O3	88771	84581	84	5	5	6	11	10
Pupa. O4	83843	79877	109	3	7	15	22	20
Pupa. O5	90087	86581	84	3	3	3	5	6
Adult. 1	87603	85355	53	2	2	2	3	3
Adult. 2	88140	85875	67	2	2	2	2	2
Adult. 3	76468	73429	146	10	9	13	16	14
Adult. 4	78755	56166	147	11	13	16	20	20
Adult. 5	79842	66193	136	9	12	16	20	15

## Data Availability

The raw data have been uploaded to the NCBI website (https://www.ncbi.nlm.nih.gov/bioproject/PRJNA1148828 accessed on 26 July 2024) using the BioProject ID PRJNA1148828.

## References

[1] Chen B., Du K., Sun C., Vimalanathan A., Liang X., Li Y., Wang B., Lu X., Li L., Shao Y. (2018). Gut bacterial and fungal communities of the domesticated silkworm (*Bombyx mori*) and wild mulberry-feeding relatives. ISME J..

[2] Xie S., Lan Y., Sun C., Shao Y. (2019). Insect microbial symbionts as a novel source for biotechnology. World J. Microbiol. Biotechnol..

[3] Kaltenpoth M., Florez L.V. (2020). Versatile and Dynamic Symbioses between Insects and *Burkholderia* Bacteria. Annu. Rev. Entomol..

[4] Lee W.J., Hase K. (2014). Gut microbiota-generated metabolites in animal health and disease. Nat. Chem. Biol..

[5] Li Y., Chang L., Xu K., Zhang S., Gao F., Fan Y. (2023). Research Progresses on the Function and Detection Methods of Insect Gut Microbes. Microorganisms.

[6] Suenami S., Koto A., Miyazaki R. (2023). Basic Structures of Gut Bacterial Communities in Eusocial Insects. Insects.

[7] Zheng H., Powell J.E., Steele M.I., Dietrich C., Moran N.A. (2017). Honeybee gut microbiota promotes host weight gain via bacterial metabolism and hormonal signaling. Proc. Natl. Acad. Sci. USA.

[8] Su L.J., Liu H., Li Y., Zhang H.F., Chen M., Gao X.H., Wang F.Q., Song A.D. (2014). Cellulolytic activity and structure of symbiotic bacteria in locust guts. Genet. Mol. Res..

[9] Shao Y., Chen B., Sun C., Ishida K., Hertweck C., Boland W. (2017). Symbiont-Derived Antimicrobials Contribute to the Control of the *Lepidopteran* Gut Microbiota. Cell Chem. Biol..

[10] Chen B., Zhang N., Xie S., Zhang X., He J., Muhammad A., Sun C., Lu X., Shao Y. (2020). Gut bacteria of the silkworm *Bombyx mori* facilitate host resistance against the toxic effects of organophosphate insecticides. Environ. Int..

[11] Philipp E., Nancy A.M. (2013). The gut microbiota of insects—Diversity in structure and function. FEMS Microbiol. Rev..

[12] Hammer T.J., Moran N.A. (2019). Links between metamorphosis and symbiosis in holometabolous insects. Philos. Trans. R. Soc. Lond B Biol. Sci..

[13] Moll R.M., Romoser W.S., Modrzakowski M.C., Moncayo A.C., Lerdthusnee K. (2001). Meconial peritrophic membranes and the fate of midgut bacteria during mosquito (Diptera: Culicidae) metamorphosis. J. Med. Entomol..

[14] Hongoh Y., Deevong P., Inoue T., Moriya S., Trakulnaleamsai S., Ohkuma M., Vongkaluang C., Noparatnaraporn N., Kudo T. (2005). Intra- and interspecific comparisons of bacterial diversity and community structure support coevolution of gut microbiota and termite host. Appl. Environ. Microbiol..

[15] Martinson V.G., Moy J., Moran N.A. (2012). Establishment of characteristic gut bacteria during development of the honeybee worker. Appl. Environ. Microbiol..

[16] Powell J.E., Martinson V.G., Urban-Mead K., Moran N.A. (2014). Routes of Acquisition of the Gut Microbiota of the Honey Bee Apis mellifera. Appl. Environ. Microbiol..

[17] Kwong W.K., Moran N.A. (2016). Gut microbial communities of social bees. Nat. Rev. Microbiol..

[18] Moran N.A., Hansen A.K., Powell J.E., Sabree Z.L. (2012). Distinctive gut microbiota of honey bees assessed using deep sampling from individual worker bees. PLoS ONE.

[19] Zheng H., Steele M.I., Leonard S.P., Motta E.V.S., Moran N.A. (2018). Honey bees as models for gut microbiota research. Lab Anim..

[20] Shapira M. (2016). Gut Microbiotas and Host Evolution: Scaling Up Symbiosis. Trends Ecol. Evol..

[21] Kueneman J.G., Gillung J., Van Dyke M.T., Fordyce R.F., Danforth B.N. (2023). Solitary bee larvae modify bacterial diversity of pollen provisions in the stem-nesting bee, *Osmia cornifrons* (Megachilidae). Front. Microbiol..

[22] Men X., Li L., Lu Z., Ouyang F., Liu L., Xu H., Yu Y. (2018). Biological characteristics and pollination service function of Maxon bee. Chin. J. Appl. Entomol..

[23] Cao Y., Zhou X., Ye B., Li L., Lu Z., Xu H., Li W., Yu Y., Men X. (2017). Analysis on the restrictive factors of the population of *Odontocheilus chinensis* in apple orchard of Shandong Province. Chin. J. Appl. Entomol..

[24] Liu L., Li L., Ouyang F., Li C., Yu Y., Qu C., Qu Z., Ye B., Men X. (2019). Fruit-Setting, Yield Increase and Economic Value Evaluation for Cherry Pollination by *Osmia excavata* Alfken in Shandong Province. Shandong Agric. Sci..

[25] Wei S.G., Wang R., Smirle M.J., Xu H.L. (2002). Release of *Osmia excavata* and *Osmia jacoti* (Hymenoptera: Megachilidae) for apple pollination. Can. Entomol..

[26] Lu H., Dou F., Hao Y., Li Y., Zhang K., Zhang H., Zhou Z., Zhu C., Huang D., Luo A. (2021). Metabarcoding analysis of pollen species foraged by *Osmia excavata* Alfken (Hymenoptera: Megachilidae) in China. Front. Ecol. Evol..

[27] Yan Z., Wang L., Reddy G.V.P., Gu S., Men X., Xiao Y., Su J., Ge F., Ouyang F. (2022). The Supercooling Responses of the Solitary Bee *Osmia excavata* (Hymenoptera: Megachilidae) under the Biological Stress of Its Brood Parasite, *Sapyga coma* (Hymenoptera: Sapygidae). Insects.

[28] Dou F.Y., Li H.Y., Song H.Y., Kou R.M., Zhou Z.Y., Luo A.R., Huang D.Y. (2022). Nesting biology of *Osmia excavata* Alfken(Hymenoptera: Megachilidae). J. Environ. Entomol..

[29] Liu W., Li Y., Lu H., Hao Y., Zhang K., Dang X., Fan X., Zhang H., Zhou Z., Zhu C. (2023). Diversity of Bacterial Communities Associated with Solitary Bee *Osmia excavata* Alfken (Hymenoptera: Megachilidae). Appl. Sci..

[30] Barbour A.B., Montgomery M.L., Adamson A.A., Díaz-Ferguson E., Silliman B.R. (2010). Mangrove use by the invasive lionfish Pterois volitans. Mar. Ecol. Prog. Ser..

[31] Magoč T., Salzberg S.L. (2011). FLASH: Fast length adjustment of short reads to improve genome assemblies. Bioinformatics.

[32] Bokulich N.A., Subramanian S., Faith J.J., Gevers D., Gordon J.I., Knight R., Mills D.A., Caporaso J.G. (2013). Quality-filtering vastly improves diversity estimates from Illumina amplicon sequencing. Nat. Methods.

[33] Edgar R.C., Haas B.J., Clemente J.C., Quince C., Knight R. (2011). UCHIME improves sensitivity and speed of chimera detection. Bioinformatics.

[34] Wang Y., Guo H., Gao X., Wang J. (2021). The Intratumor Microbiota Signatures Associate with Subtype, Tumor Stage, and Survival Status of Esophageal Carcinoma. Front. Oncol..

[35] Xiang X., Zhang F., Fu R., Yan S., Zhou L. (2019). Significant differences in bacterial and potentially pathogenic communities between sympatric hooded crane and greater white-fronted goose. Front. Microbiol..

[36] Rubin B.E.R., Sanders J.G., Turner K.M., Pierce N.E., Kocher S.D. (2018). Social behaviour in bees influences the abundance of *Sodalis* (Enterobacteriaceae) symbionts. R. Soc. Open Sci..

[37] Dale C., Welburn S.C. (2001). The endosymbionts of tsetse flies: Manipulating host-parasite interactions. Int. J. Parasitol..

[38] McFrederick Q.S., Mueller U.G., James R.R. (2014). Interactions between fungi and bacteria influence microbial community structure in the *Megachile rotundata* larval gut. Proc. R. Soc. Biol. Sci..

[39] Hu C., Rzymski P. (2022). Non-Photosynthetic *Melainabacteria* (Cyanobacteria) in Human Gut: Characteristics and Association with Health. Life.

[40] Zang J., Xu Y., Xia W., Yu D., Gao P., Jiang Q., Yang F. (2018). Dynamics and diversity of microbial community succession during fermentation of Suan yu, a Chinese traditional fermented fish, determined by high throughput sequencing. Food. Res. Int..

[41] Huang S., Sheng P., Zhang H. (2012). Isolation and identification of cellulolytic bacteria from the gut of *Holotrichia parallela* larvae (Coleoptera: Scarabaeidae). Int. J. Mol. Sci..

[42] Xu L., Lu M., Xu D., Chen L., Sun J. (2016). Sexual variation of bacterial microbiota of *Dendroctonus valens* guts and frass in relation to verbenone production. J. Insect Physiol..

[43] Briones-Roblero C.I., Hernández-García J.A., Gonzalez-Escobedo R., Soto-Robles L.V., Rivera-Orduña F.N., Zúñiga G. (2017). Structure and dynamics of the gut bacterial microbiota of the bark beetle, *Dendroctonus rhizophagus* (Curculionidae: Scolytinae) across their life stages. PLoS ONE.

[44] Wang M., Xiang X., Wan X. (2020). Divergence in Gut Bacterial Community Among Life Stages of the Rainbow Stag Beetle *Phalacrognathus muelleri* (Coleoptera: Lucanidae). Insects.

[45] Gutierrez C., Somoskovi A. (2014). Human Pathogenic Mycobacteria. Ref. Modul. Biomed. Sci..

[46] Martha G., Stephen L.B., Brenda J.L. (1984). Microbial flora of the larval provisions of the solitary bees, *Centris pallida* and *Anthophora* sp. Apidologie.

[47] Zhang Z., Jiao S., Li X., Li M. (2018). Bacterial and fungal gut communities of *Agrilus mali* at different developmental stages and fed different diets. Sci. Rep..

[48] Yun J.H., Roh S.W., Whon T.W., Jung M.J., Kim M.S., Park D.S., Yoon C., Nam Y.D., Kim Y.J., Choi J.H. (2014). Insect gut bacterial diversity determined by environmental habitat, diet, developmental stage, and phylogeny of host. Appl. Environ. Microbiol..

[49] Engel P., Martinson V.G., Moran N.A. (2012). Functional diversity within the simple gut microbiota of the honey bee. Proc. Natl. Acad Sci. USA.

[50] Michener C.D. (1974). The Social Behavior of the Bees: A Comparative Study.

